# Humans gradually integrate sudden gain or loss of visual information into spatial orientation perception

**DOI:** 10.3389/fnins.2023.1274949

**Published:** 2024-01-08

**Authors:** Jamie Voros, Victoria Kravets, Kieran Smith, Torin K. Clark

**Affiliations:** Ann and H.J. Smead Department of Aerospace Engineering Sciences, Boulder, CO, United States

**Keywords:** spatial orientation, disorientation, pilot, orientation perception, visual orientation, vestibular, vestibular orientation

## Abstract

**Introduction:**

Vestibular and visual information is used in determining spatial orientation. Existing computational models of orientation perception focus on the integration of visual and vestibular orientation information when both are available. It is well-known, and computational models capture, differences in spatial orientation perception with visual information or without (i.e., in the dark). For example, during earth vertical yaw rotation at constant angular velocity without visual information, humans perceive their rate of rotation to decay. However, during the same sustained rotation with visual information, humans can continue to more accurately perceive self-rotation. Prior to this study, there was no existing literature on human motion perception where visual information suddenly become available or unavailable during self-motion.

**Methods:**

Via a well verified psychophysical task, we obtained perceptual reports of self-rotation during various profiles of Earth-vertical yaw rotation. The task involved transitions in the availability of visual information (and control conditions with visual information available throughout the motion or unavailable throughout).

**Results:**

We found that when visual orientation information suddenly became available, subjects gradually integrated the new visual information over ~10 seconds. In the opposite scenario (visual information suddenly removed), past visual information continued to impact subject perception of self-rotation for ~30 seconds. We present a novel computational model of orientation perception that is consistent with the experimental results presented in this study.

**Discussion:**

The gradual integration of sudden loss or gain of visual information is achieved via low pass filtering in the visual angular velocity sensory conflict pathway. In conclusion, humans gradually integrate sudden gain or loss of visual information into their existing perception of self-motion.

## Introduction and motivation

The sensory input can be broken down into passive and active inputs across several sensory organs, where the activation of passive sensory receptors differs from actively activating sensory organs (Gibson, 1983; Stoffregen et al., 2017). Passive experience of motion is inherently contrived because humans use active motion to explore (and thus perceive) their environment (Mantel et al., 2015) and perception is influenced by external factors (such as balance) (Riccio et al., 1992). The passive sensory input in the visual and vestibular channels has previously been shown to be sufficient for predicting orientation perception during some motion paradigms (Benson, 1978; Merfeld et al., 1993; Newman, 2009; Merfeld, 2017). Accurate perception of orientation is associated with the successful motor control of crewed vehicles such as aircraft and spacecraft. Periods of disorientation may present a major threat to smooth operation of manually controlled aerospace vehicles (Bellenkes et al., 1992; Young et al., 2003; Dixon et al., 2019). For example, the operation of aircraft in meteorological conditions (visual cues are degraded) is associated with a high incidence of fatal mishaps (Ayiei et al., 2020). Given the potentially disastrous consequences of spatial disorientation [class A mishaps and fatality (Dixon et al., 2019)], it is important that we understand when disorientation is likely during flight-like motions. By measuring orientation perception during motions which is possible in a laboratory setting, it is possible to build and validate models of orientation perception (Merfeld et al., 1993, 1999; Newman, 2009; Clark et al., 2019; Williams et al., 2021). Models of orientation perception allow us to simulate dynamic motions which are not possible in a laboratory setting and make informed inferences about whether or not a motion could induce disorientation during flight. By identifying motions that may result in spatial disorientation, it may be possible to trigger countermeasures (Dixon et al., 2019, 2022) or other approaches (Dixon et al., 2019) and ultimately achieve higher levels of success in manually controlled piloting. It is important to note, however, that the manual operation of aerospace vehicles involves a combination of passive and active motion. Measuring and modeling passive motion (the aim of the current study) is a necessary precursor to building models appropriate for flight scenarios which include active control.

## Orientation perception modeling background

Mathematical models that dynamically integrate sensory cueing (such as vestibular, visual, or somatosensory information) exist (Selva and Oman, 2012; Clark et al., 2019). Such models have been developed in an effort to better understand how the central nervous system integrates sensory cueing (Clark et al., 2019). Some models of orientation perception are built upon estimation theory concepts (MacNeilage et al., 2008), such as Kalman filter (Kalman, 1960) models (Laurens and Angelaki, 2017), particle filter models (Laurens and Droulez, 2007; Karmali and Merfeld, 2012; Kravets et al., 2021; Allred et al., 2023), and non-linear models (*Observer* models) (Selva and Oman, 2012). *Observer* models are non-linear models of spatial orientation perception that, as a key function, use sensory conflict to predict perception (Luenberger, 1971; Oman, 1982; Merfeld et al., 1993; Merfeld and Zupan, 2002; Zupan et al., 2002; Vingerhoets et al., 2007, 2009; Newman, 2009; Rader et al., 2009; Williams et al., 2021). *Observer* models hypothesize that the central nervous system contains an internal model of sensory systems and orientation perception that is used to generate expected sensory afference. The expected sensory afference is compared to true sensory afference. The difference (sensory conflict) is fed back into the model to update the internal model of orientation perception. Kalman filter models (Borah et al., 1988; Selva and Oman, 2012) use “optimal weighting” of the sensory conflict (i.e., the innovation) based upon measurement and processing noise, solving the Riccati equation, as opposed to the “tuned weighting” gains in the observer. While there are different formulations, particle filters use resampling to estimate distributions and thus do not require the assumption of normality. The parallel particles have been considered analogous to the many afferent neuron measurements (Laurens and Droulez, 2007; Karmali and Merfeld, 2012).

Older *Observer* models of orientation perception tend to focus on orientation perception as understood via vestibular cues only (Ormsby and Young, 1976; Borah et al., 1977; Oman, 1991; Merfeld et al., 1993; Vingerhoets et al., 2007, 2009). Some *Observer* models of orientation perception accommodate multisensory (visual and vestibular) cuing (Newman, 2009; Clark et al., 2019). Based on existing models of visual and vestibular perception in the presence of visual information that are congruent to true motion, a human observer is generally able to perceive their motion fairly accurately (Guedry and Lauver, 1961; Newman, 2009; Vingerhoets et al., 2009; Fetsch et al., 2012; Laurens and Angelaki, 2016; Dixon et al., 2019). However, unlike vestibular cues which are always present in an observer with a healthy vestibular system, visual information may not be present for an entire dynamic motion. For example, during flight, flying into and out of clouds is commonplace (for pilots with an instrument rating). When flying into a cloud, the pilot experiences a sudden disappearance of visual information. Similarly, upon flying out of a cloud, the pilot experiences the opposite. While existing models of orientation perception have begun to incorporate visual cueing pathways, no existing model of orientation perception robustly accounts for a sudden change in the availability of visual orientation information despite this being a common flight scenario.

Here, we aimed to quantify the dynamic time course of self-rotation perception during transitions in the availability of visual information (becoming available or unavailable). Next, we will enhance an existing computational model of spatial orientation perception to mimic the empirical perceptual time course following these visual transitions.

## Experimental methods

This study was approved by the University of Colorado’s Institutional Review Board under Protocol #19-002. Each subject signed an informed consent form prior to participation.

### Subjects

Seventeen subjects participated (7 female, mean age 30 SD ± 5 years; 15 whites and 2 more than one race) but not all subjects completed the full course of testing. Exclusion criteria were self-reported history of vestibular dysfunction, age outside 18–40 years (Bermúdez Rey et al., 2016), and a motion sickness susceptibility questionnaire above the 90th percentile. Subjects requiring glasses for 20–20 vision were excluded because the HMD was not compatible with glasses. In total, three subjects did not complete testing as a result of feeling cybersickness during the experiment.

### General experimental description

Subjects were rotated in Earth-vertical yaw motion and asked to report their perception of angular velocity. This motion paradigm isolates the semicircular canals of the vestibular system because it does not alter the stimulation to the otoliths. During motions, the availability of visual information was manipulated. Visual information was suddenly removed during some motions and suddenly gained during others. We refer to whether or not visual information was available (or the availability suddenly changed) as “visual information availability conditions.”

**Figure 1:**
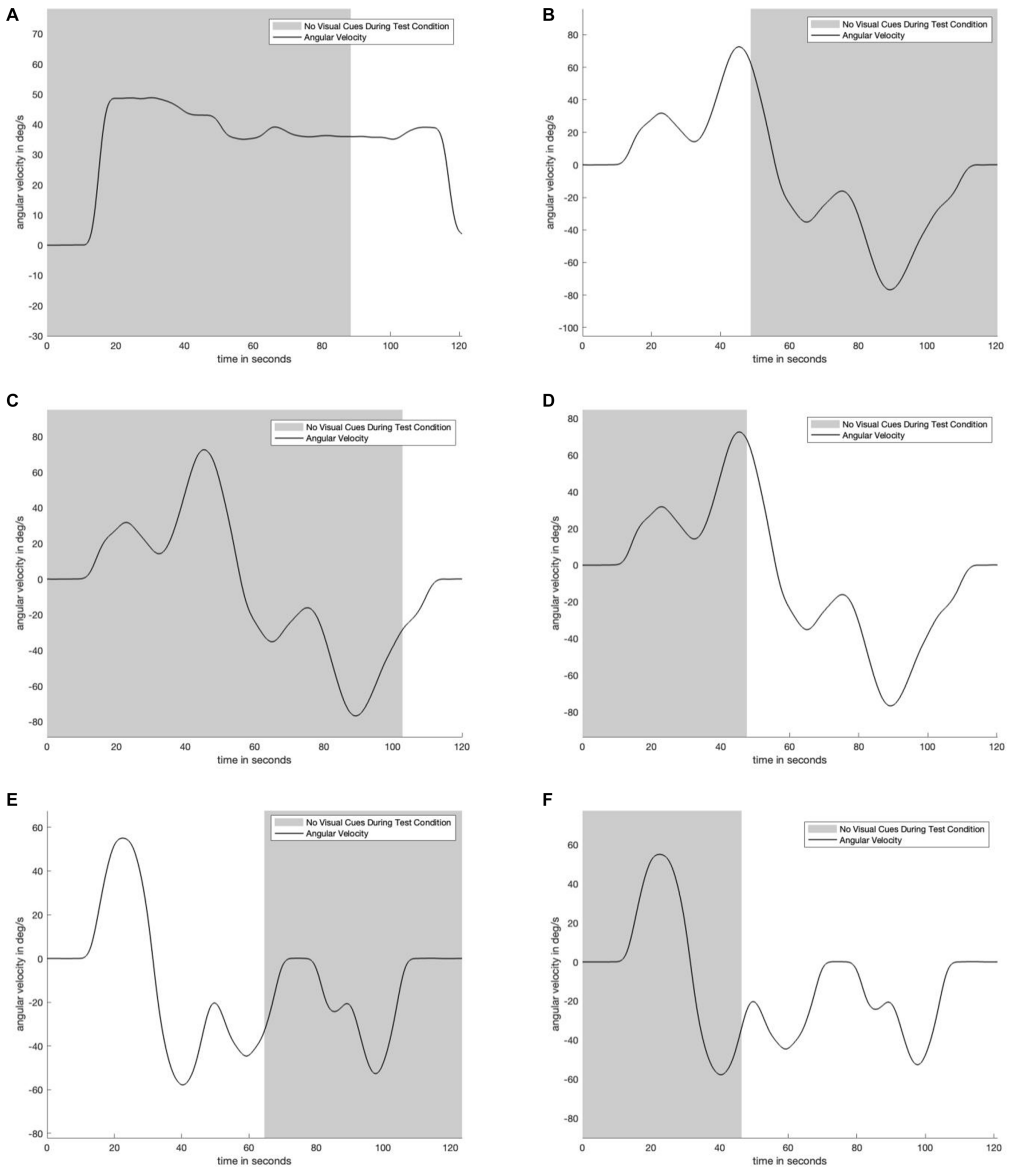
Plot matrix to show underlying motion profiles and visual information availability used during testing. Panel **(A)** shows the unidirectional motion profile in which for the test condition, visual information suddenly appeared after 88 seconds. Panel **(B)** shows a bidirectional motion profile in which for the test condition visual information suddenly disappeared after 49 seconds. Panel **(C)** shows the same motion profile as in panel **(B)**, but where the visual information suddenly appeared after 103 seconds. Panel **(D)** is the same motion profile as panels **(C)** and **(D)**, but where the visual information suddenly appeared after 47 seconds. Panels **(E)** and **(F)** show the same bidirectional motion profile, but in panel **(E)** the visual information suddenly disappears after 64 seconds, while in panel **(F)** the visual information suddenly appears after 45 seconds. Five of six motion profiles are bidirectional and have near constant change in angular velocity to reduce motion predictability. The shaded area indicates when subjects were and were not provided with visual angular velocity information.

We refer to a “motion profile” as an approximately 2-min-long period in which subjects experienced yaw rotation to the left, right, or both. Some motion profiles also contained stationary periods (i.e., zero angular velocity). Figure 1 depicts one of the motion profiles used in this study. Subjects experienced three unique motion profiles throughout the course of the study. By comparing across visual information availability conditions on each of the same motion profiles, we aimed to quantify the dynamic time course of self-rotation perceptions resulting from transitions in the availability of visual cues.

### Test and control conditions

There were four visual information availability conditions:

1. Visual information available for the entire motion (control)
2. Visual information suddenly disappears part way through the motion (test)
3. Visual information suddenly becomes available part way through the motion (test)
4. No visual information available throughout the motion (i.e., in the dark) (control)

**Figure 2:**
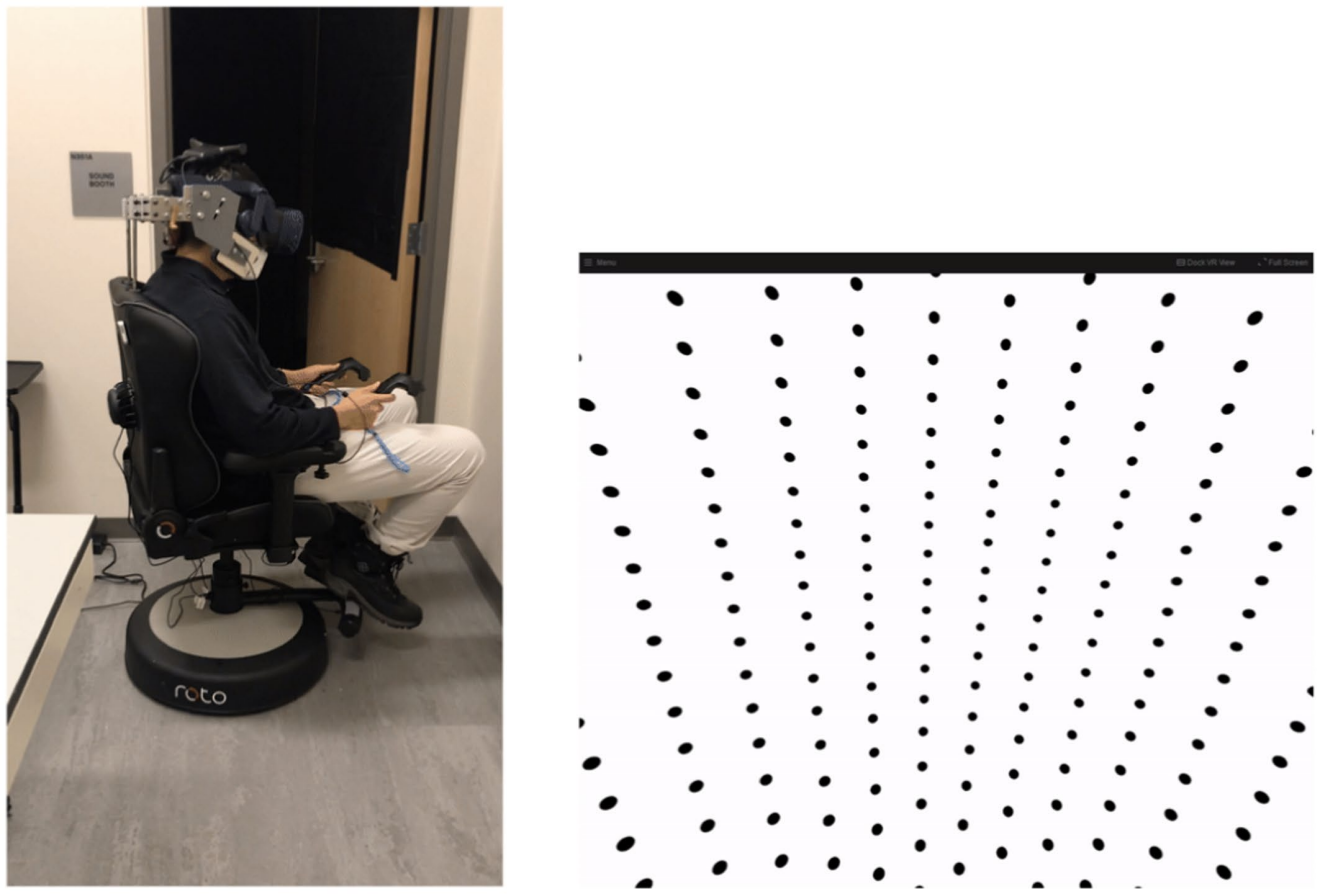
Left: Photograph to show experimental apparatus. Testing was conducted in the dark and with the rear door closed (lights on for photograph). Right: Visual display provided to subjects when visual information were made available. The dot pattern moved in the opposite direction of rotation to provide congruent angular visual velocity information.

Conditions (1) and (4) were control conditions and served to evaluate these methods against existing studies. Conditions (2) and (3) served as test conditions during which we aimed to quantify perception of self-rotation following the sudden gain or loss of visual information. Visual information was provided in the form of a dot pattern that would move congruent to the true rotation experienced by subjects, as shown in Figure 2. When no visual information was provided, the head-mounted display (HMD) was black. In order to mitigate the impacts of stray light within the HMD, data collection occurred in a dark room. In addition, subjects experienced white noise both through auditory (via headphones) and tactile (via chair vibration) sensory channels in order to mask potential spurious sensations of motion outside the vestibular and visual channels. White noise as provided via headset headphones was kept constant by keeping the volume settings (and apparatus) consistent throughout testing. White noise was provided via a rumble pack producing chair vibration. Auditory white noise was approximately 60 dB, while chair vibration was approximately +/−0.46 m/s^2 SD. Figure 2 shows the experimental setup before the lights were turned out for testing.

Each subject experienced each motion profile at most four times, each time under a different visual information availability condition. Each motion profile and visual information availability condition combination is called a “trial.” About half of the subjects experienced positive angular velocity as rotation to the right and the other half experienced positive angular velocity as rotation to the left. The assignment of positive angular velocity direction was randomized per subject per motion profile. The random assignment of the direction of positive angular velocity was done in order to account for potential differences between left and right angular velocity perception. Based on qualitative examination of subject responses, we found no substantial difference in the perception of self-rotation to the right vs. self-rotation to the left. In order to collate aggregate subject perceptions, subjects who experienced positive angular velocity as rotation to the left had their responses “flipped”: their perceptions of self-rotation were multiplied by −1.

### Apparatus

Subjects were seated in a chair that rotated about an Earth-vertical yaw axis (RotoVR, Borehamwood, UK) (see Figure 2). The chair was adapted to include a head restraint to ensure that subjects’ head motion (and thus stimulation to the vestibular organs) was consistent with chair motion (see Figure 2). Visual information was provided via wireless virtual reality (VR) head mounted display (HMD) (HTC, New Taipei City, Taiwan). When provided, the visual information was always congruent with true motion. Subjects held two controllers (HTC, New Taipei City, Taiwan), one in each hand in order to report perception of motion. The controllers had thumbpads (beneath the thumb) and rear triggers (beneath the index finger).

### Visual information

Within the HMD, subjects were “inside” a large sphere with a dot pattern on the inside, as shown in Figure 2, right. Note that the view is prior to distortion applied when the image passes through the lenses of the HMD. The visual scene provided optical flow but did not include any information regarding Earth-horizontal or angular position (e.g., azimuth).

### Psychophysical task

Subjects reported their dynamic perceptions of yaw motion by pressing the thumbpad of the controller in their left/right hand every time they felt like they had rotated 90 degrees left/right. Subjects held the triggers on the back of the controllers if they felt they were not moving. The task of indicating rotation every 90 degrees has been employed and validated previously (Groen and Jongkees, 1948; Guedry and Lauver, 1961; Voros and Clark, 2023).

### Procedure

Subjects completed four practice trials and had the option to complete more (but none obliged). Subjects then completed each trial in a randomized order. Subjects were able and encouraged to take breaks (and remove the HMD) during testing. Subjects were asked to rate their sleepiness and cybersickness after each trial. Sleepiness was rated on a scale of 1 to 9, 1 being fully alert and 9 being actively fighting sleep. Cybersickness was reported as a binary “yes” or “no” response. If a subject reported feeling cybersick for three trials in a row, their testing session was terminated. This is because we did not want to cause subjects discomfort and because our goal was to collect high-quality data.

### Data processing

Perceived angular velocity was computed by dividing 90 degrees by the time between each button press (or time between button press and trigger release/hold start). This was assumed to be angular velocity perception for the entire duration between two button presses. Each subject’s perception of angular velocity was computed at each discretized timestep; from this, the mean and standard error were computed to produce aggregate angular velocity perception across all subjects. A Gaussian window filter was applied (3 s on each side) to smooth aggregate data. Lastly, we anticipated that subjects would accurately perceive motion when visual information was provided, with only slight adjustments to account for how subjects reported perception using the psychophysical task. Therefore, subject data from the control condition where visual information was always provided was used to set an angular velocity scale factor for the psychophysical task used. The scale factor that best fit all subject data was 1.15, meaning that subjects’ perception of angular velocity when visual information was provided was consistent with true angular velocity. A scale factor was necessary to account for how the psychophysical task translates to perception: although the task is inherently perceptual, we did not assume that subjects would accurately associate the verbal instructions of “press the button every 90 degrees” to 90 degrees in the physical world.

## Experimental results

**Figure 3:**
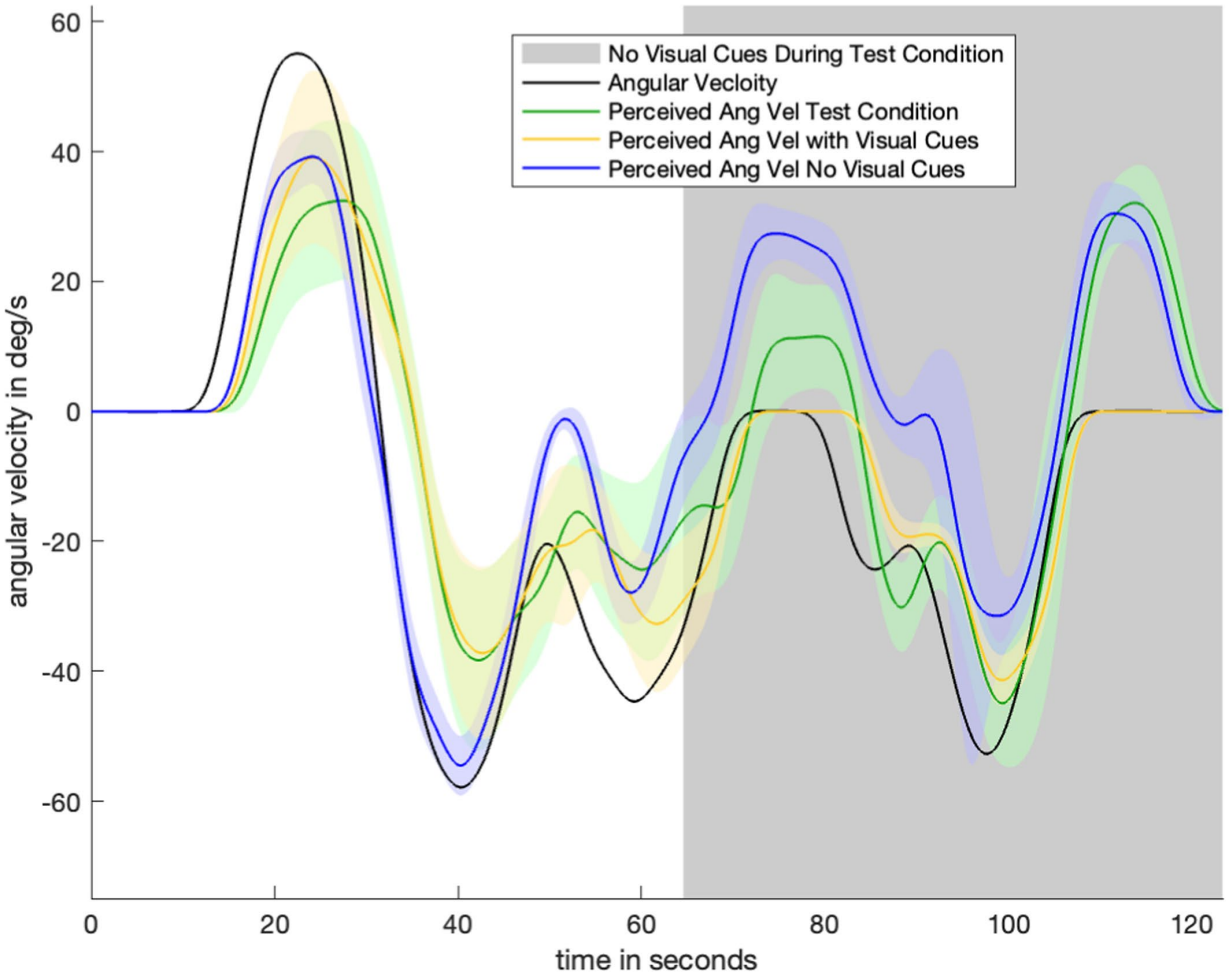
Plot to show experimental data for one motion profile-test condition combination (Figure 1E). Visual information/without visual information control conditions for that motion profile are shown in navy/yellow. Green is average orientation perception for the test condition. Notably, the green line begins by closely tracking the yellow (with visual information control condition) line. After the visual information is suddenly removed (gray part of plot), the green line transitions to tracking the navy (no visual information control condition) line.

Figure 3 shows data for one test condition and two control conditions across one motion profile. Average perception during the test condition (green) begins by closely tracking the “with visual information” control condition (yellow). After the visual information is suddenly removed (gray), perception during the test condition (green) transitions to tracking the “no visual information” control condition (navy). There is a 30-s transitionary period between the removal of visual information and the test condition (green) becoming similar to (and within the standard error bounds of) the second control condition (navy). Notably, angular velocity perception during the transitionary period (immediately after visual information was suddenly removed) is somewhere between angular velocity perception in the two control conditions. At nearly 70 s, during the stationary period, there is misperception in the opposite direction but not to the same extent of the misperception during the no-visual-information (navy) control condition. This pattern of perception indicates that the subjects gradually adjusted their perception in accordance with the new lack of visual information. Despite the same stimulus having been delivered to the vestibular organs, subjects do not immediately report the same perception after visual information was removed as when visual orientation information was never provided at all.

**Figure 4:**
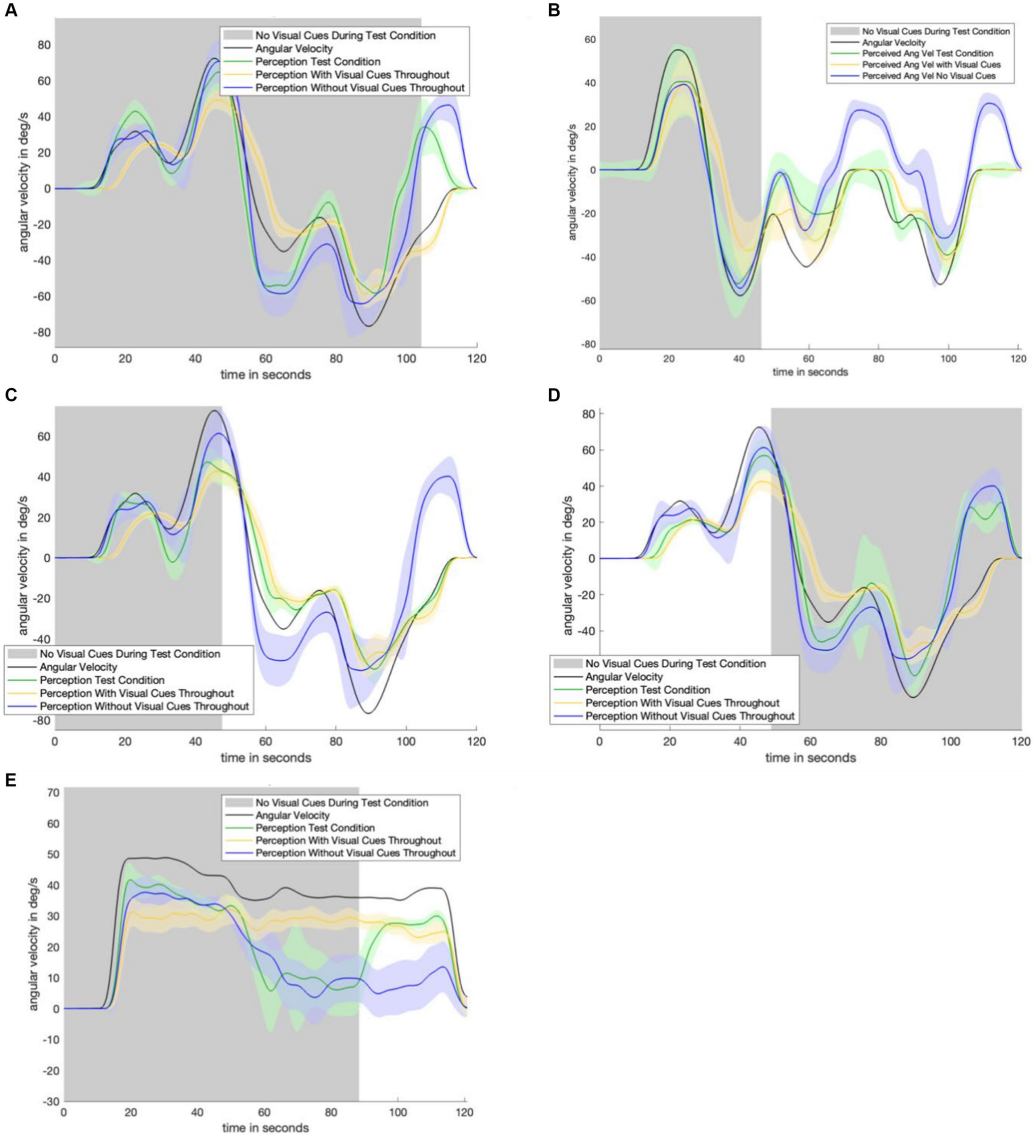
Remaining experimental data. Shaded areas of each plot indicate where visual information was not provided during the test condition. Each plot contains data from the control conditions. Navy is the control condition with no visual information, yellow is the control condition with visual information. Green is the test condition. Panel **(A)** shows the results from the motion profile in Figure 1C. Panel **(B)** shows the results from the motion profile in Figure 1C. Panel **(C)** shows the results from the motion profile in Figure 1D. Panel **(D)** shows the results from the motion profile in Figure 1B. Panel **(E)** shows the results from the motion profile in Figure 1A.

Figure 4 displays the perceptual data in the remaining motion profiles and visual transitions. The top four panels show the sudden appearance of visual information. There is a transition period of approximately 10 s: it takes approximately 10 s for the test condition (green) line to transition from following the no visual information condition (navy) line to following the with visual information (yellow) line. Figures 3 4D show a 30-s transition period in the opposite direction (where visual information is suddenly removed). Figure 3 shows that the impact of velocity storage resulting in the sensation of motion in the opposite direction after one has become stationary is reduced. Notably, both a 10-s and 30-s delay are longer than the delay expected to be associated with the psychophysical perceptual task. With a button press every 90 degrees and subjects rotating by approximately 40 degrees per second, it would take less than 3 s (90 degrees/40 degrees/s < 3 s) for perception to “jump” from a decayed state to being consistent with the actual motion (in the case of visual information suddenly appearing) if the delay were only a matter of the psychophysical perceptual task.

## Modeling methods

The sudden addition of information in the visual angular velocity pathway is modeled as a step input. The step response of a low-pass filter is a gradual rise of the output signal. The data indicated that our subjects gradually integrated the new (or new loss of) visual information. Therefore, adding a low pass filter to the visual angular velocity pathway was a prime candidate for reconciling the existing observer model with the subject data. A low pass filter has a gain and time constant associated with it. There was an existing gain within the visual angular velocity pathway leaving the time constant (of the low pass filter) as the only new free parameter.

Using average angular velocity perception against time, it was possible to quantitatively fit model parameters (gains and filter time constants). Root mean squared error (RMSE) as calculated at each discretized timestep between average subject angular velocity perception and model-predicted angular velocity perception was used as the cost function for parameter fitting. Model parameters were set by using Fminsearch (MATLAB, 2006) to minimize RMSE via varying parameter selection.

The control condition where no visual information was provided stimulates the vestibular angular velocity pathway only. Therefore, subject data from the control condition without visual information was used to set $k_{\omega }$ , and the gain was associated with vestibular angular velocity perception via the semicircular canals. $k_{\omega }=25$ best fit our data. Notably, $k_{\omega }=25$ is higher than the gain of $k_{\omega }=8$ which was used in the original (Newman, 2009), model. However, the (Newman, 2009), model set $k_{\omega }=8$ based on Vingerhoets et al. (2006). Vingerhoets performed a small search over six potential $k_{\omega }$ values, of which 8 was the highest they considered. It is possible that a $k_{\omega }>8$ may have fit Vingerhoets’ data better, but the research group did not test any. In order to avoid a multi-variable optimization process between $k_{\omega v}$ and our added parameter $\tau_{\omega v},$ we set $k_{\omega v}=k_{\omega }$ . Lastly, we ran a single variable optimization to fit $\tau_{\omega v}$ , the time constant of the newly added low pass filter. We fitted the model to just four of our six test conditions so that we could then compare model prediction to two unseen test conditions afterward. $\tau_{\omega v}=5.0$ s minimized RMSE between the four test conditions used to train the model and also qualitatively matched the two test conditions previously unseen.

As an aside, we found the model predictions matched the empirical data with visual information available similarly well with $k_{\omega v}$ values that were roughly similar to $k_{\omega }$ (e.g., doubling or halving $k_{\omega v}$ yielding similarly good fits). As our goal was not to optimize $k_{\omega v}$ , we chose to set it to simply be equal to $k_{\omega }.$ We also did not find substantial differences in $\tau_{\omega v}$ values that minimized RMSE with changes in $k_{\omega v}$ and $k_{\omega }$ . A further investigation of nuisance parameter setting ( $k_{\omega v}$ , $k_{\omega }$ ) is elaborated upon in the discussion.

## Modeling results

**Figure 5:**
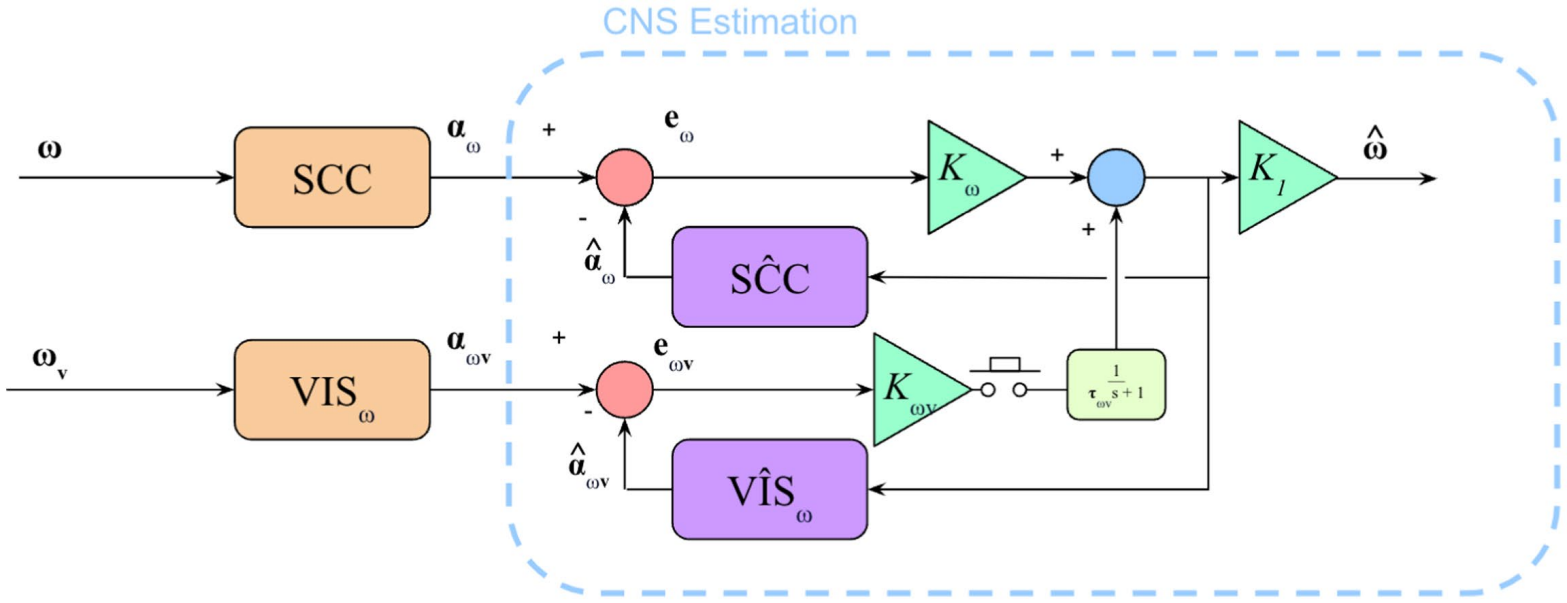
Model to show semicircular canal and visual angular velocity pathways. Additional parts of the model exist (e.g., otolith sensing, visual position and vertical) but only the SCCs and visual angular velocity pathway were being stimulated in this experiment. The addition of the low pass filter (in yellow) in the visual angular velocity sensory conflict pathway captures the gradual integration of sudden loss or gain of visual information.

**Figure 6:**
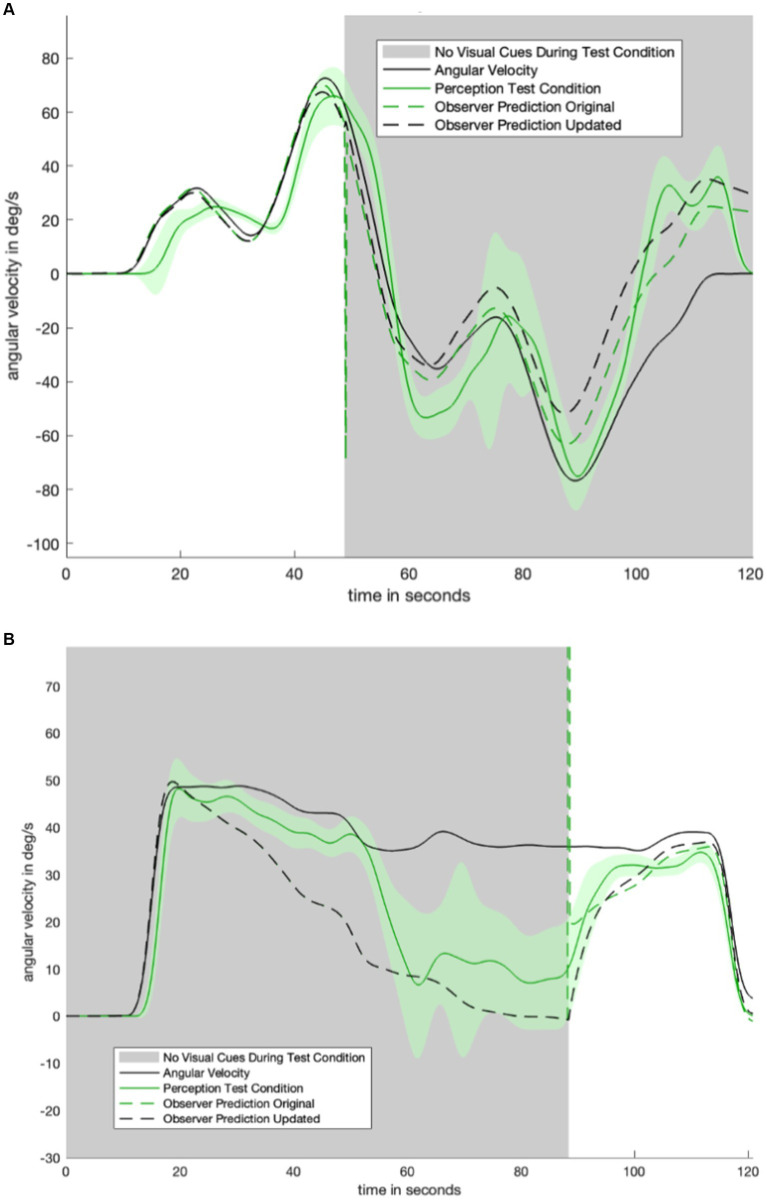
Two trials which were not used in fitting model parameters. In black is the predicted perception of the updated model. We note that addition of an appropriately tuned low pass filter removes the discontinuity seen in the original mode (dashed green). Panel **(A)** shows the model-predicted perception for the motion profile in Figure 1B, with the corresponding empirical perception from the test condition shown in Figure 4D. Panel **(B)** shows the model-predicted perception from the motion profile in Figure 1A, with the corresponding empirical perception from the test condition shown in Figure 4E.

Running the newly parameterized model of orientation perception (Figure 5) on the two trials previously unseen by the model resulted in predicted perception that was qualitatively consistent with experimental data. Figure 6 compares the original (Newman, 2009) model (i.e., without the low pass filter added to the visual angular velocity sensory conflict pathway) to the new model proposed in this study.

The new model (dashed black line, Figure 6) qualitatively matches the characteristic shape of the perceptual data from study participants. As shown in Figure 6, the discontinuity which appears at the visual transition is resolved in the updated model.

## Discussion

To summarize, we performed a human psychophysical experiment to quantify the time course of self-rotation perception during transitions in the availability of visual information. We found that visual information was integrated gradually, such that when they became available, the resulting perception of self-rotation converged toward that when visual information was always available over the time course of ~10 s. When visual information was removed, the dynamic perception slowly transitions over approximately 30 s. We incorporated these empirical findings into an enhanced computational model of spatial orientation perception.

### Experimental findings

**Figure 7:**
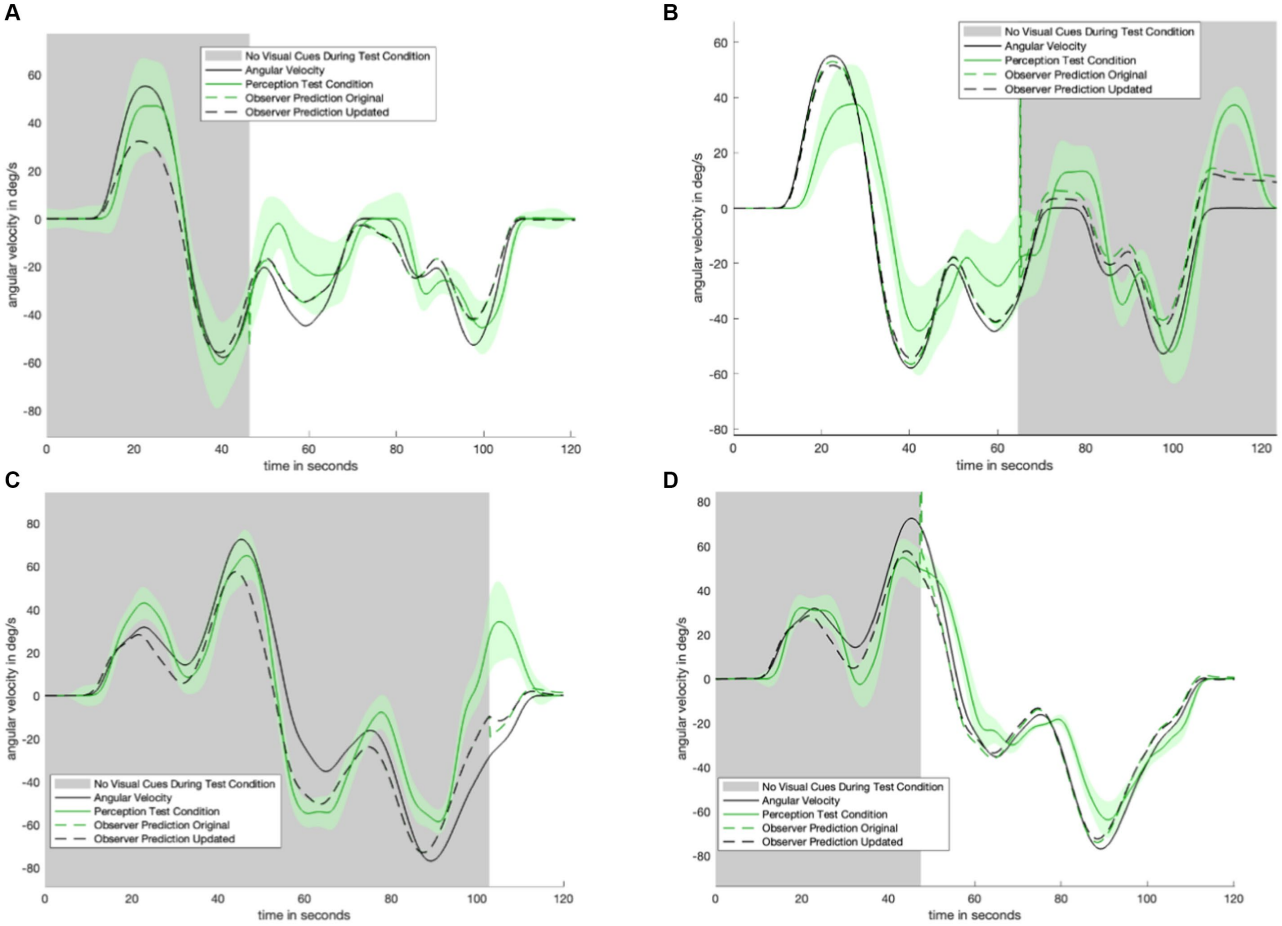
Plot matrix to show original model prediction against updated model prediction for remaining trials. The data (shown in solid green) was used to fit the parameters of the updated model. Panel **(A)** shows the model-predicted perception for the motion profile in Figure 1F, with the corresponding empirical perception from the test condition shown in Figure 4B. Panel **(B)** shows the model-predicted perception for the motion profile in Figure 1E, with the corresponding empirical perception from the test condition shown in Figure 3. Panel **(C)** shows the model-predicted perception for the motion profile in Figure 1C, with the corresponding empirical perception from the test condition shown in Figure 4A. Panel **(D)** shows the model-predicted perception for the motion profile in Figure 1D, with the corresponding empirical perception from the test condition shown in Figure 4C.

A limitation of this study is the use of virtual reality to deliver visual information. While the visual information was congruent with subjects’ true motion, existing literature indicates that virtual visual orientation cueing may not necessarily be equivalent to visual cues found in the physical world (Ivanenko et al., 1998; Kimura et al., 2017). However, for rotational cues in particular, virtual visual information still delivers a robust sense of angular motion (Pretto et al., 2009). Additionally, during motions where no visual information was provided, there is less decay than the model predicts. In Figure 6, the dashed line (model prediction) decays (becomes lower) faster than the solid green line (average subject perception). It is possible that slower perceptual decay occurs because the motion profile is highly predictable. In particular, large differences in decay are not seen in the other, more variable, motion profiles shown in Figure 7. Another limitation is that subject perceptions were highly variable. Average subject perception is shown as a solid line throughout this study. Notably, however, the standard error shown on either side is often substantial enough that in some cases subjects were perceiving angular velocity in opposite directions (for example, between 60 and 80 s of the profile shown in Figure 6B). The high variability of angular velocity perception can be attributed to the blunt nature of the psychophysical task. Subjects only gave input every 90 degrees. For slower motions, this means that there could be up to several seconds between button presses. However, as noted earlier, the delay associated with the task is substantially smaller than the time taken to gradually integrate new gain or loss of visual information. Therefore, the gradual integration of visual information (or new loss of visual information) is not solely due to the psychophysical task.

The novel model of orientation, based on data collected in this study, is designed to be representative of pilots. Typically, pilots are physically healthy and with no known visual or vestibular dysfunction. The subject pool of the present study was screened for healthy visual and vestibular function and was limited to participants under the age of 40 years to account for changes in vestibular sensing due to age. As such, the model of orientation presented is applicable to the target population. Therefore, it may be possible to use the updated model of orientation perception to assess the potential for spatial disorientation during flight scenarios that could involve a sudden gain or loss of visual information. Being able to determine if a flight maneuver may result in spatial disorientation is instrumental for mission design. For example, crafting a safe landing sequence (which does not induce disorientation) is critical for the success of crewed space missions to other planetary bodies.

Combinations of linear accelerations and angular motions are more typical during flight than yaw motion alone (which occurs during rotor wing flight). A limitation of this study is the examination of yaw motion alone. However, it is necessary to understand what happens to orientation perception during sudden visual transition in the base case (isolated angular motion) before examining more complex motions. Future work could build on this study and examine orientation perception across visual transitions during more complex motions. For example, visual horizontality information (in the case of roll tilt) coupled with linear acceleration may not be gradually integrated into perception the same way sudden (and sudden loss of) visual angular velocity information is.

### Computational model enhancement

**Figure 8:**
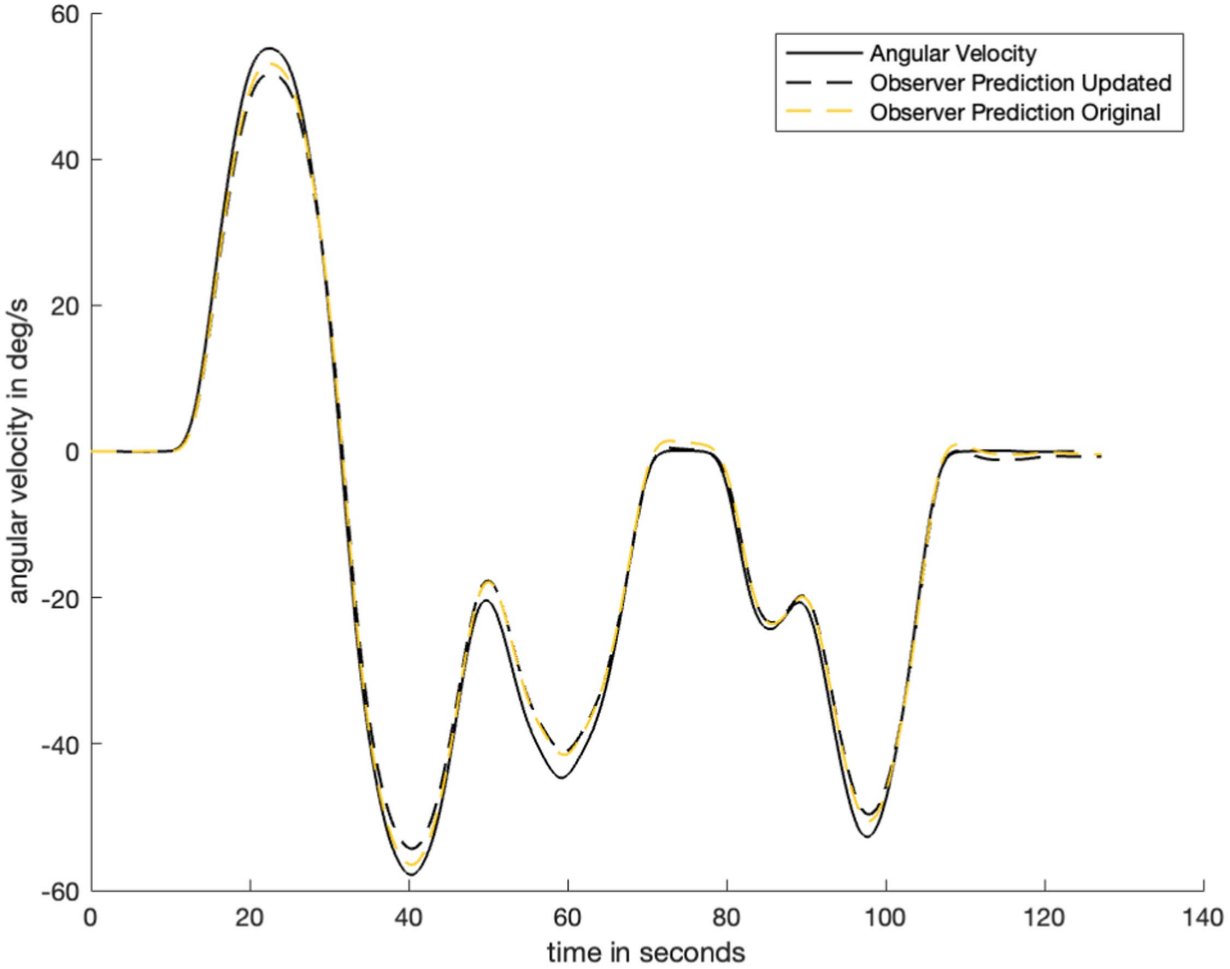
Plot to show difference in observer model prediction with the addition of the low pass filter in the visual angular velocity pathway. Here, we note that the addition of low pass filtering does not substantially change the observer prediction when the visual information availability condition does not change. Therefore, the updated model is still valid for cases where the previous model was valid (e.g., perception predictions with visual angular velocity information available).

The parameter fitting process presented in the current study is more quantitative (Merfeld et al., 1993) and robust (Vingerhoets et al., 2007) than methods used previously. However, raising $k_{\omega }$ beyond 25 yields an unstable perception prediction. Additionally, a quantitative parameter fitting process was not performed for $k_{\omega v}$ because this was not the primary purpose of the study. The assumption that $k_{\omega }=k_{\omega v}$ is not inherently correct but opens the door for future research examining the relationship (or differences) between the two parameters. Additionally, the quantitative fitting process was performed for $k_{\omega }\neq k_{\omega v}$ . With a $k_{\omega v}=12$ (Newman, 2009), the time constant of the novel low pass filter, $\tau_{\omega v}$ , converged to 5.3 s. Thus, small changes to $k_{\omega }$ and $k_{\omega v}$ still result in a fitted $\tau_{\omega v}$ of the same order of magnitude. The addition of filtering to the visual angular velocity pathway does not substantially change previously verified predictions of orientation perception. Figure 8 shows that the model prediction (dashed lines) is not substantially different before and after the addition of low pass filtering in the visual angular velocity pathway. As such, the model presented in this study is still consistent with existing literature on angular velocity perception when visual information is always provided. There is no difference between the (Newman, 2009) model and the model presented in this study in scenarios where visual information is never provided because the only change was made to a visual model pathway: The part of the model that was updated is not used when visual information is never present.

The addition of a low pass filter aptly captures perceptual dynamics following a sudden transition in the availability of visual information. However, it is possible that alternative modeling strategies could capture the gradual change in perceptual patterns observed in the empirical data presented in this study. Particle filtering or dynamic reweighting of sensory information both have potential to explain the perceptual dynamics captured in our data. Orientation perception models currently predict a single perception for a given dynamic scenario. However, our experimental data indicates some level of variability in subjects’ perception during motion. Furthermore, variability in subject responses appears greater in the absence of visual information. Future modeling efforts could change model architecture to include both predicted perception and expected variability in perception.

While our modeling approach was able to capture the empirical data responses fairly well, including in unseen test data, we acknowledge that there may be other, yet to be proposed, alternative modeling approaches that could explain the data equally well, or even better. Future studies should consider potential alternative modeling approaches as well as empirically quantify a broader range of motion profiles to serve as additional test data for model validation.

Additionally, existing literature indicates that active motion is used in perceiving orientation (Mantel et al., 2015) and that environmental dynamics impact perception (Stoffregen and Riccio, 1988). For future modeling efforts to be truer to the underlying scientific characteristics of orientation perception, future work must focus on incorporating the active control and dynamics of the specific aerospace vehicle being controlled. The model as it stands, however, has potential to serve as a flight planning or training tool: disorientation simulators can increase flight safety (Boril et al., 2016). In order to build a disorientation simulator, it is necessary to identify motions that may result in disorientation which this model is capable of.

## Conclusion

There has been no prior quantification of motion perception during a sudden transition in the availability of visual information. Through the sudden removal or provision of visual information at critical moments during angular motion, it was possible to quantify how perception changes immediately following a transition. The data indicates that as opposed to immediately accepting new visual information or immediately relying on vestibular information only, humans gradually integrate the new information (or new loss of information) into their perception of orientation.

Existing models of orientation were unable to aptly quantify perception of angular motion when visual information suddenly appeared or disappeared. Here, we present a model of orientation perception that is both robust to sudden changes in the availability of visual information and consistent with experimental data. The model makes use of a low pass filter to model the gradual integration of visual angular velocity information seen in the subject data.

Lastly, we present a quantitative method of model parameterization for this class of observer models. Quantitatively identifying model parameters marks a step forward in orientation perception modeling as, previously, model parametrization had been done by hand.

## Data availability statement

The original contributions presented in the study are included in the article/supplementary material, further inquiries can be directed to the corresponding author.

## Ethics statement

The studies involving humans were approved by the Institutional Review Board at the University of Colorado Boulder. The studies were conducted in accordance with the local legislation and institutional requirements. The participants provided their written informed consent to participate in this study. Written informed consent was obtained from the individual(s) for the publication of any potentially identifiable images or data included in this article.

## Author contributions

JV: Conceptualization, Data curation, Formal analysis, Investigation, Methodology, Software, Visualization, Writing – original draft, Writing – review & editing. VK: Conceptualization, Investigation, Methodology, Software, Writing – review & editing. KS: Conceptualization, Investigation, Methodology, Software, Writing – review & editing. TC: Conceptualization, Formal analysis, Funding acquisition, Investigation, Methodology, Project administration, Resources, Supervision, Validation, Writing – review & editing.
